# Synthesis of Mesoporous γ-Al_2_O_3_ with Spongy Structure: In-Situ Conversion of Metal-Organic Frameworks and Improved Performance as Catalyst Support in Hydrodesulfurization

**DOI:** 10.3390/ma11071067

**Published:** 2018-06-24

**Authors:** Dandan Liu, Hongwei Zhu, Jinchong Zhao, Longjun Pan, Pengcheng Dai, Xin Gu, Liangjun Li, Yunqi Liu, Xuebo Zhao

**Affiliations:** 1 Research Centre of New Energy Science and Technology, Research Institute of Unconventional Oil & Gas and Renewable Energy, China University of Petroleum (East China), Qingdao 266580, China; liudandan_upc@126.com (D.L.); dpcapple@upc.edu.cn (P.D.); guxin@upc.edu.cn (X.G.); lilj@upc.edu.cn (L.L.); 2 State Key Laboratory of Heavy Oil Processing, China University of Petroleum (East China), Qingdao 266580, China; zhuhongweiyh@126.com (H.Z.); dr.zhaojc@gmail.com (J.Z.); longjunpan@126.com (L.P.)

**Keywords:** alumina, metal-organic frameworks, microstructure, hydrodesulfurization, catalyst support

## Abstract

Over the past decades, extensive efforts have been devoted to modulating the textural properties, morphology and microstructure of γ-Al_2_O_3_, since the physiochemical properties of γ-Al_2_O_3_ have close correlations with the performance of hydrotreating catalysts. In this work, a spongy mesoporous γ-alumina (γ-Al_2_O_3_) was synthesized using Al-based metal-organic frameworks (Al-MOFs) as precursor by two-step pyrolysis, and this Al-MOF-derived γ-Al_2_O_3_ was used as hydrodesulfurization (HDS) catalyst support, to explore the effect of support on the HDS performance. Compared with industrial γ-Al_2_O_3_, the spongy alumina displayed well-developed porosity with relatively high surface area, large pore volume, and abundant weak Lewis acid sites. Based on catalyst characterization and performance evaluation, sulfurized molybdenum and cobalt molecules were able to incorporate and highly disperse into channels of the spongy mesoporous alumina, increasing the dispersion of active catalytic species. The spongy γ-Al_2_O_3_ was also able to enhance the diffusion efficiency and mass transfer of reactant molecules due to its improved texture properties. Therefore, the corresponding catalyst presented higher activities toward HDS of dibenzothiophene (DBT) than that from industrial alumina. The spongy mesoporous γ-alumina synthesized by Al-MOFs provides a new alternative to further develop novel γ-alumina materials with different texture and various nanoporous structures, considering the diversity of MOFs with different compositions, topological structures, and morphology.

## 1. Introduction

To meet the increasing emphasis on quality upgrading requirements of clean fuels, it is imperative to improve and develop hydrotreating catalysts with high activity [1,2]. Activated alumina, especially γ-Al_2_O_3_, has been widely used as supports in commercial catalysts for hydrotreating and petroleum refining processes. High surface area, porous structure, suitable acidity, as well as excellent thermal and mechanical stability of γ-Al_2_O_3_ are all beneficial factors for catalyst supports [3,4]. Besides, the physiochemical properties of alumina have close correlations with the morphology and dispersion of the supported active phase, which affects catalytic performance, such as activity and selectivity. Hence, controlling of textural properties, morphology and microstructure of γ-Al_2_O_3_ are important aspects to improve the efficiency of alumina in catalysis [5]. Generally, the structure and morphology of γ-Al_2_O_3_ could be inherited from the precursor during the thermal transformation process. Therefore, complete understanding of precursor and innovative synthesized procedures are essential to synthesize γ-Al_2_O_3_ with excellent performance.

The acquirement of γ-Al_2_O_3_ is mostly through the thermal dehydration of (pseudo-)boehmite in a different phase at 400–750 °C [6,7]. The traditional γ-Al_2_O_3_ thus manufactured may contain non-negligible amounts of alkali ions, which affect the activity and lifetime of active phase, and present texture properties with relatively low surface area (less than 250 m^2^/g) and limited pore volume (less than 0.5 cm^3^/g) determined by the stacking of primary particles. Boehmite with high purity could be synthesized as a coproduct through Ziegler process, while the formation of intermediates, aluminum trialkyls, involves the hydrogenation of aluminum powder at higher pressure (10–20 MPa), which causes high energy consumption and requires high quality equipment. Hence, it is necessary to devote efforts to the synthesis of pure alumina with convenient methods and more penetrable textural property. In the past decade, a new porous material, Al-based metal-organic frameworks (Al-MOFs), has been attracting considerable interest in academic as well as industrial research [8,9,10]. Al-MOFs are built from aluminium inorganic vertices, most often metal-oxo-clusters, which are connected with each other by organic ligand. In the structure of Al-MOFs, various Al-oxo clusters have been reported, which include trimeric and octameric clusters [11,12,13], one-dimensional chains of corner- or edge-sharing Al(OH)_2_O_4_-octahedra [14,15], as well as octanuclear and dodecameric cyclic clusters [16,17], offering a large number of alternative precursors for alumina. To explore the potential of various aluminium clusters, the organic ligand could be totally eliminated by thermal transformation, forming alumina assembled by different aluminium clusters with high purity. The study of Al-MOFs as a precursor to fabricate γ-Al_2_O_3_ has not aroused much attention. Based on our previous study, aluminium clusters in Al-MOFs could direct the microstructure and morphology of the obtained alumina [18,19,20]. Therefore, using Al-MOFs as precursor is a route that may expand the versatility of alumina.

Herein, to further explore the synthetic diversity of γ-Al_2_O_3_, we developed a facile approach to prepare mesoporous γ-Al_2_O_3_ with spongy structure by two-step pyrolysis of an Al-MOFs (Al(OH)(1,4-NDC)_2_·H_2_O, noted Al-NDC). The framework of Al-NDC was constructed by the infinite Al(OH)_2_O_4_ chains linked through hydroxyl groups, which are interconnected by the 1,4-naphthalenedicarboxylate (1,4-NDC) groups [21]. During the two-step pyrolysis treatment of Al-NDC, the position of ligand was vacated and avoiding the aggregation of the Al-OH-Al chains, forming γ-Al_2_O_3_ with spongy-like structure. As the hydrodesulfurization (HDS) catalyst support, the large surface area and weak Lewis acidic sites of spongy γ-Al_2_O_3_ enhance the dispersion of active phase; the preliminary catalytic evaluations show that the catalyst using spongy γ-Al_2_O_3_ as support presents superior performance than that using commercial γ-alumina in HDS reaction to remove dibenzothiophene (DBT).

## 2. Materials and Methods

### 2.1. Synthesis of Al-MOFs

In this study, the Al-MOFs was synthesized via a hydrothermal method mainly according to the reference [21]. Al(NO_3_)_3_∙9H_2_O (1.5 g, 4.0 mmol) and organic ligand 1,4-naphthalene-dicarboxylate acid (noted 1,4-H_2_NDC, 0.432 g, 2.0 mmol) was added into 40 mL deionized water, and then the mixture was transferred into an 100 mL Teflon autoclave, which was allowed to react at 180 °C for 24 h. The precipitate was filtered off and washed with deionized water for several times, then dried in an oven at 80 °C for 8 h. The obtained light-yellow powder was recorded as Al-NDC.

### 2.2. Synthesis of Al_2_O_3_ Support

The Al-MOFs precursor was treated under two-step pyrolysis as follows. Firstly, the obtained Al-NDC precursor was placed in a tube furnace and the tube was flushed three times by high-purity nitrogen to remove air. Then, the Al-NDC was annealed at 900 °C for 3 h in nitrogen, with a heating rate of 3 °C min^−1^. Afterwards, the intermediate product was transferred into muffle furnace after cooling to room temperature. Porous Al_2_O_3_ were obtained by calcining in air from room temperature to 500 °C at a rate of 5 °C∙min^−1^, and maintained at this temperature for 4 h. The obtained γ-Al_2_O_3_ using Al-MOFs as precursor by two-step pyrolysis was denoted as Al_2_O_3_-Spongy.

To compare with Al_2_O_3_-Spongy, a γ-Al_2_O_3_ using commercial pseudo-boehmite as precursor was obtained as reference. The conversion of pseudo-boehmite (Shandong Alumina Plant, Zibo, China) into γ-Al_2_O_3_ was achieved by calcination in muffle furnace. The pseudo-boehmite powder were calcined at 500 °C for 4 h at a rate of 5 °C∙min^−1^, and a white product was obtained, denoted as Al_2_O_3_-PB.

### 2.3. Preparation of CoMo/Al_2_O_3_ Catalyst

The catalysts were prepared using incipient wetness impregnation, and the obtained oxidation-state supports were co-impregnated with mixed aqueous solution containing (NH_4_)_6_Mo_7_O_24_∙4H_2_O and Co(NO_3_)_3_∙6H_2_O. The loading amounts of MoO_3_ and CoO were controlled to be 17.3 wt % and 2.7 wt %, respectively. The impregnated supports were then dried in air overnight, before calcinating at 500 °C in muffle furnace for 4 h. The obtained catalysts using Al_2_O_3_-Spongy and Al_2_O_3_-PB as support were denoted as Catalyst-Spongy-O and Catalyst-PB-O, respectively.

### 2.4. Characterization of Materials and Catalysts

X-ray powder diffraction (XRD) measurements of precursor and catalysts were performed using a X’Pert PRO MPD X-ray diffractometer (Panalytical, Almelo, Netherlands) equipped with Cu Kα radiation (λ = 1.5406 Å) (40 kV, 40 mA) at scan rate of 0.257°·s^−1^ in 2θ. Inductively coupled plasma atomic emission spectroscopy (ICP-AES) was measured by a Thermo ICAP 7000 spectrometer (Thermo Fisher Scientific, Waltham, MA, USA). The sample was mixed with lithium tetraborate and heated at 925 °C for 15 min, then the residue was dissolved in boiling tartaric acid- hydrochloric acid aqueous solution, and diluted to 50 mL with water.

All scanning electron microscopy (SEM) images were recorded on a Hitachi S-4800 field-emission SEM microscope (Hitachi Ltd., Chiyoda, Tokyo, Japan). The samples for SEM analysis were fixed on a support using electric conductive adhesive, coated with a layer of gold. Transmission electron microscopic (TEM) investigations and high-resolution transmission electron microscopy (HRTEM) analysis were carried out with a JEOL JEM-2100UHR (JEOL Ltd., Akishima, Tokyo, Japan). Samples were ultrasonic dispersed in ethanol, then dropped on a carbon-coated Cu grids for the TEM analysis, and 300 MoS_2_ slabs were measured to analyse the average slab length and stacking number distribution. 

Pyridine fourier transform infrared (FT-IR) spectra of alumina samples were recorded on a Bruker 4700 FT-IR spectrometer (Bruker Optics Inc., Ettlingen, Germany) with a liquid-nitrogen- cooled MCT detector. Prior to measurement, the samples were dehydrated in muffle furnace at 350 °C for 3 h, then transferred in the saturated pyridine atmosphere overnight after cooling down. After adsorption, the pyridine by physical adsorption was eliminated in vacuum oven at 110 °C for 3 h. By using Kubelka-Munk pattern, the obtained FT-IR diffuse reflection spectra was able to transform into liquid-like spectra to minimise specular reflection.

The textural properties of the alumina samples were measured by nitrogen physisorption isothermals at 77 K on Quantachrome Autosorb-iQ nitrogen volumetric adsorption instrument (Quantachrome Instruments, Boynton Beach, FL, USA). All gas sorption isotherms were measured after pretreatment under a dynamic vacuum at 300 °C overnight.

H_2_-temperature-programmed reduction (H_2_-TPR) of the sulfurized catalysts was conducted with a Micromeritics AutoChem 2950 instrument (Micromeritics Instrument Corp., Norcross, GA, USA). Before reduction, the 0.1 g sample was pretreated in Ar at 150 °C for 2 h to remove the adsorbed water, then the Ar flow was switched to 10% H_2_/Ar flow, and the catalyst was heated to 600 °C at a rate of 10 °C min^−1^.

The NH_3_-termperature-programmed desorption (NH_3_-TPD) curves of obtained alumina were also recorded by a Micromeritics AutoChem 2950 instrument (Micromeritics Instrument Corp., Norcross, GA, USA). Prior to NH_3_ adsorption, 0.1 g sample was pretreated in Ar at 400 °C for 2 h, then cooled down to 80 °C, and Ar flow was switched to NH_3_/He flow until the sample is saturated with NH_3_. The adsorbed NH_3_ by physisorption was removed by purging of He for 1 h. Finally, the catalyst was heated to 600 °C at a rate of 10 °C min^−1^ to desorb the NH_3_ by chemisorption.

X-ray photoelectron spectroscopy (XPS) was performed on a Thermol Scientific ESCALAB 250Xi spectrometer (Thermo Fisher Scientific, Waltham, MA, USA) equipped with a monochromatic Al Ka (E = 1486.6 eV) radiation.

### 2.5. Hydrodesulfurization Catalytic Activity Assessment

The hydrodesulfurization (HDS) catalytic activity assessments were evaluated in a 100 mL stainless steel batch reactor. Before evaluation, oxidation-state catalysts were ex-situ sulfurized under a flow of (100 mL·min^−1^) H_2_S/H_2_ (10:90 by volume) at 400 °C for 4 h, and the obtained sulfurized catalysts were named as Catalyst-Spongy-S and Catalyst-PB-S. Then 40 g (0.55 wt % DBT dissolved in decalin) of reactant was transferred into the reactor, accompanied with 0.4 g of the sulfurized catalyst. Prior to reaction, the reactor was purged three times with H_2_ to replace the air inside and then pressurized to 2.0 MPa at room temperature. The reaction was performed at 300 °C under stirring at a rate of 600 r min^−1^ for 8 h, and the reaction pressure reached 7.2 MPa. The liquid product was analyzed by an Agilent 7890 GC instrument (Agilent Technologies Inc., Santa Clara, CA, USA) equipped with a flame ionization detector (FID) and a HP-5 Agilent alumina capillary column.

The initial HDS kinetic constants (*k_HDS_*) were calculated assuming pseudo-first order kinetics by Equation (1) [22]:

(1) $$k_{HDS}=\frac{n_{DBT}}{t\times m_{cat}}ln\left(\frac{1}{1-x_{DBT}}\right)$$

in which *n_DBT_* represents the moles of DBT in the feed, *m_cat_* is the catalyst mass in Kg, *x_DBT_* is the conversion of DBT which is below 40%, *t* is the reaction time in second, and *k_HDS_* is the rate constant of HDS in mol Kg_cat_^−1^ s^−1^.

Based on the method developed by Kastzelan [23,24], the MoS_2_ dispersion was calculated by the number of Mo atoms located at the side (noted as *Mo_side_*) and the total number of Mo atoms (noted as *Mo_total_*) according to the Equation (2):

(2) $$f_{Mo}=\frac{Mo_{side}}{Mo_{total}}=\frac{\sum_{i=1}^{t}\left(6n_{i}-6\right)}{\sum_{i=1}^{t}\left(3n_{i}{}^{2}-3n_{i}+1\right)}$$

where *n_i_* is the number of Mo atoms along the side of a MoS_2_ slab calculated from the length (L = 3.2(2n_i_ − 1) Å), and *t* is the total number of slabs in the TEM images.

The HDS turnover frequency (TOF) was estimated by *f_Mo_* via Equation (3):

(3) $$\mathrm{TOF}=\frac{n_{DBT}\times x_{DBT}}{t\times n_{Mo}\times f_{Mo}}$$

where *n_DBT_* represents the moles of DBT in mol, *x_DBT_* is the DBT conversion, *n_Mo_* is the amount of Mo atoms in the catalyst in mol, and *t* is the reaction time in hour.

Selectivity of the direct desulfurization (DDS) route and the hydrogenation (HYD) route was calculated according to the HDS reaction network of DBT. The product of DDS route is biphenyl (BP), while the products through HYD pathway are cyclohexylben-zene (CHB) and bicyclohexyl (BCH), as well as traces of tetrahydrodibenzothiophene (4H-DBT), hexahydrodibenzothiophene (6H-DBT). Therefore, HYD/HDS selectivity was calculated based on Equation (4):

(4) $$S_{\mathrm{HYD}/\mathrm{DDS}}=\frac{C_{CHB}+C_{BCH}+C_{4H-DBT}+C_{6H-DBT}}{C_{BP}}$$

where *C_CHB_*, *C_BCH_*, *C_4H-DBT_*, *C_6H-DBT_* and *C_BP_* are the concentrations of CHB, BCH, 4H-DBT, 6H-DBT and BP in the reaction products, respectively.

## 3. Results and Discussion

### 3.1. Characterization of γ-Al_2_O_3_ Synthesized from Al-MOFs

**Figure 1:**
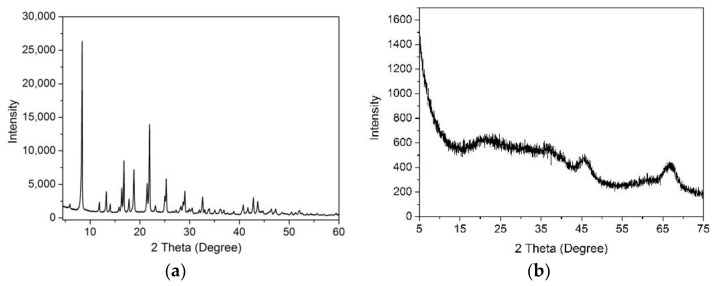
XRD patterns of (**a**) Al-MOFs precursor and (**b**) the obtained γ-Al_2_O_3_.

The XRD patterns of the Al-NDC precursor and obtained alumina by two-step pyrolysis are displayed in Figure 1. The diffraction peaks attributing to Al-NDC are consistent with the reported pattern [21]. After the two-step pyrolysis treatment, the diffraction peaks attributing to Al-NDC disappear, indicating a complete decomposition of original frameworks. The obtained pattern of thermal treatment product can be well indexed to the phase of γ-Al_2_O_3_ (JCPDS 00-050-0741), where two characteristic diffraction peaks at 45.7° and 66.7° are assigned to the (400) and (440) planes of γ-Al_2_O_3,_ respectively, and no residual carbon was detected in the final γ-Al_2_O_3_. The weak and broadening diffraction peak at about 22° indicates the mere existence of amorphous alumina. The low intensity and broadened diffraction peaks suggest that the product is poor in crystallinity and assembled by small crystallites. Through the chemical analysis of Al_2_O_3_ derived from MOFs (Table 1), only traces of Na_2_O, Fe_2_O_3_ and SiO_2_ could be detected, indicating high purity of the alumina derived from Al-MOFs.

**Figure 2:**
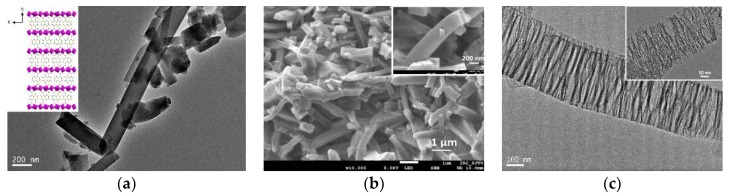
TEM images of (**a**) Al-MOFs precursor, insert is the structure of Al-MOFs (Al-1,4-NDC), Al-NDC; (**b**) SEM images and (**c**) TEM images of obtained Al_2_O_3_-Spongy by two-step pyrolysis of Al-NDC, inset is the magnified TEM and SEM images of Al_2_O_3_-Spongy.

**Scheme 1:**
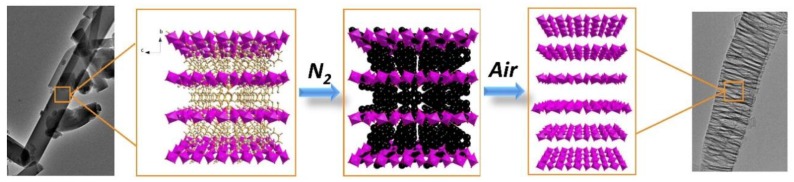
The transformation process from Al-NDC precursor to Al_2_O_3_-Spongy by two-step pyrolysis.

To characterize the detailed morphology and microstructure of Al-MOFs precursor and the obtained γ-Al_2_O_3_ by two-step pyrolysis, the morphology and interior structure were carefully observed by SEM and TEM. As shown in Figure 2a, the precursor appears in rod-like morphology with smooth surface, and no obvious pores are detected in the internal structure. After the two-step pyrolysis, the obtained Al_2_O_3_ particles retained their original size and rod-like morphology of the MOF precursor. More interesting, the curved alumina fiber possesses nanoporous structures, and the surface of products becomes rough and is decorated with obvious voids and slit pores (Figure 2b). The inner structure are then reflected by TEM images (Figure 2c), which found that the obtained Al_2_O_3_ presents spongy-like structure (denoted as Al_2_O_3_-Spongy). Numerous alumina lamina with average thickness of 3.98 nm results in the formation of slit-like pores throughout the alumina fiber, and these pores had access to the outside, offering abundant channels and surfaces for molecules to enter and pass through. The generation of this structure is mainly due to deterioration and carbonization of the alternating organic naphthalene layers [21], which agrees with the layered crystalline structure of the MOFs precursor (Scheme 1). The two-step pyrolysis method is beneficial for preserving the configuration of Al-NDC. Firstly, the in-situ carbonization of ligand at high temperature in nitrogen could act as a temporal buffer preventing the contraction and aggregation of aluminium chains in Al-MOFs, and the high temperature could also ensure the thermal stability of alumina. Then, the carbon was eliminated in air to vacate the original space of ligand, resulting in the formation of γ-Al_2_O_3_ with spongy-like structure accompanied by the release of CO_2_.

**Figure 3:**
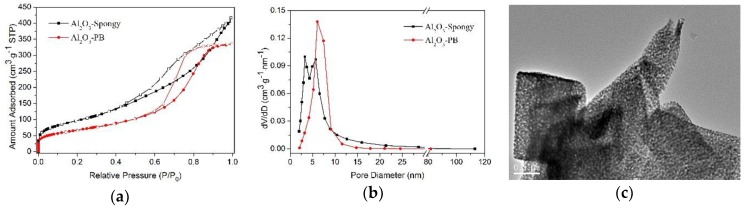
(**a**) N_2_ adsorption-desorption isotherms and (**b**) pore size distribution of Al_2_O_3_-Spongy and Al_2_O_3_-PB; (**c**) TEM images of commercial alumina Al_2_O_3_-PB.

The porous structure of the as-prepared Al_2_O_3_-Spongy was examined by N_2_ sorption isotherms at 77 K, and BJH (Barrett-Joyner-Halenda) pore size distribution analysis was conducted. To identify the advantages of Al_2_O_3_-Spongy, an alumina sample derived from commercial pseudo-boehmite (denoted as Al_2_O_3_-PB) was obtained as reference on the same calcination conditions; the corresponding texture properties of Al_2_O_3_-Spongy and Al_2_O_3_-PB are listed in Table 2. As shown in Figure 3a, the obtained Al_2_O_3_-Spongy displays a type IV adsorption isotherm, which is characterized by the mesopores in the interior structure. An H3-type hysteresis loop over a relative pressure range of 0.42 < P/P_0_ < 1.0 is observed, attributing to the slit-like pores stacked by laminar structure. The type of isotherm and hysteresis loop agrees well with the TEM observations and the spongy-like structure with uniform slit-like pores in Al_2_O_3_-Spongy. The BET surface area of Al_2_O_3_-Spongy is 350 m^2^ g^−1^, and the pore volume is 0.63 cm^3^ g^−^^1^, both higher than reported alumina materials synthesized by boehmite precursor and other Al-MOFs [18,19,20,25,26]. The N_2_ sorption isotherms of Al_2_O_3_-PB are also Type IV (Figure 3a), suggesting the mesoporous structure of this alumina. According to IUPAC classification, the hysteresis loop of the isotherm for Al_2_O_3_-PB is H2 type, which confirms the existence of ink bottle pores stacked by uniform particle size. This hysteresis loop is in agreement with the inner structure of Al_2_O_3_-PB (Figure 3c), which is assembled by numerous uniform secondary particles in the range of 30–45 nm. The mesopore size distributions of the obtained Al_2_O_3_ were calculated using BJH method (Figure 3b). The pore size distribution curve of Al_2_O_3_-Spongy exhibits a bimodal distribution in the range of 2–16 nm, and the two peak-tops located at 3.2 nm and 5.7 nm may correspond to the entrance of channel and lamina distance respectively. Pore size distribution of Al_2_O_3_-PB is concentrated between 2–15 nm, and a centered peak at 6 nm is detected. The relative high BET surface area, pore volume and two peak-tops pore distributions of obtained Al_2_O_3_-Spongy material are mainly brought about by the spongy-like structure with laminar nanosheets.

**Figure 4:**
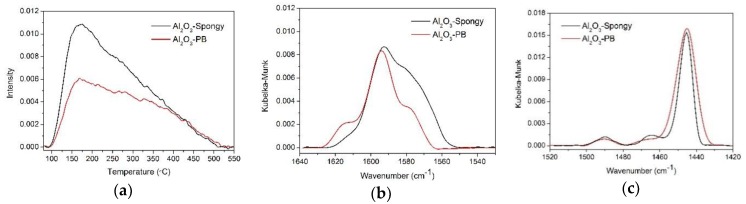
(**a**) NH_3_-TPD profiles obtained from Al_2_O_3_-Spongy and Al_2_O_3_-PB; and (**b**,**c**) DRIFT spectra of pyridine adsorption on the obtained alumina samples.

The acidic properties of alumina originated from surface hydroxyl groups and Al^3+^ derived from dehydration, and the acidity of supports may have an effect on improving the dispersion of active metals and even increasing activity [27]. Figure 4a shows the NH_3_-TPD profiles of the obtained γ-alumina samples, to reveal the amount of acid sites on alumina surface. Both of the TPD patterns present an intense peak in the range of 100–500 °C, and the intensity of peak reflects the amount of adsorbed NH_3_ on the surface acidic sites. In general, the detected acidic sites by NH_3_-TPD can be conventionally divided into weak (100 °C < T < 300 °C), moderate (300 °C < T < 500 °C) and strong (T > 500 °C) [28]. Al_2_O_3_-Spongy presents higher desorption peak at lower-temperature range than Al_2_O_3_-PB, indicating higher ratio of weak acid sites on the surface of Al_2_O_3_-Spongy. However, the area of desorption peak attributing to moderate acid presents little difference between Al_2_O_3_-Spongy and Al_2_O_3_-PB. Therefore, based on the NH_3_-TPD results, Al_2_O_3_-Spongy reported in the present study possesses more weak surface acidic sites compared to commercial alumina Al_2_O_3_-PB.

In order to characterize surface acidity, pyridine was used as the probe molecule, whose vibration bands reflect the nature and strength of the acidic sites [29]. Figure 4b,c show the IR spectra of pyridine (Py) adsorbed on alumina surface in the range of 1640–1420 cm^−1^ after heating the samples at 110 °C under vacuum for 2 h. The two alumina samples both display five bands at 1614 cm^−1^, 1593 cm^−1^, 1579 cm^−1^, 1490 cm^−1^ and 1445 cm^−1^. The vibrations of pyridine ring could be divided into 8a (1630–1585 cm^−1^), 8b (1579 cm^−1^), 19a (1490 cm^−1^), and 19b (1445 cm^−1^) [30], and different bands in Py-IR are used to evaluate the Lewis and Bronsted acid sites on the surface. The bands at 1614 cm^−1^ is assigned to pyridine coordinately bonded to Lewis acid sites of moderate strength; the bands at 1593 and 1445 cm^−1^ are due to the hydrogen bonded pyridine; 1579 cm^−1^ corresponding to weak Lewis acid sites bonded pyridine, and the band at 1490 cm^−1^ can be assigned to pyridine bonded to both Lewis and Bronsted sites. From the area under the curve, it can be found that Al_2_O_3_-Spongy possesses more weak Lewis acid sites than Al_2_O_3_-PB, while the amount of moderate acid sites are basically similar, and this result is in accordance with NH_3_-TPD. This may be relative to the two-step pyrolysis treatment of Al-MOFs. The in-situ carbonization of ligand could protect the original Al-OH-Al chains in Al-MOFs and prevent their aggregation at the first stage. While during the second step treatment in air, compared with oxygen atoms from oxygen, the elimination of carbon will preferentially separate the oxygen atoms from Al(OH)_2_O_4_ octahedra, resulting in more weak Lewis acid sites on alumina surface. Therefore, the two-step pyrolysis is helpful to enhance the concentration of acidic sites, especially weak Lewis acidic sites on the surface of Al_2_O_3_-Spongy.

### 3.2. Characterization of CoMo/Al_2_O_3_ Catalysts

**Figure 5:**
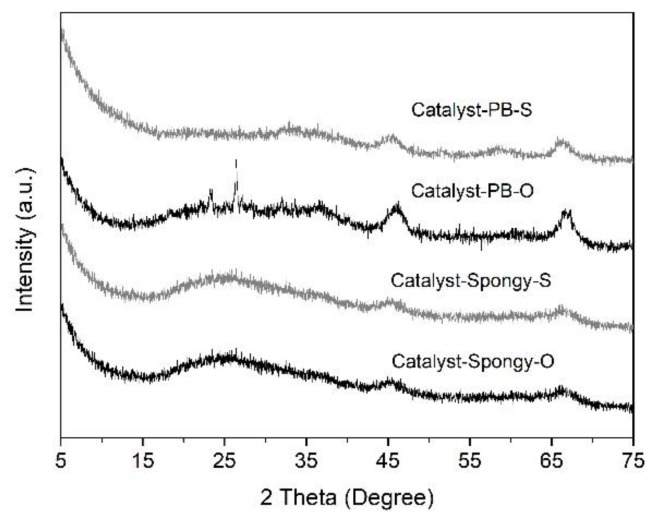
XRD patterns of the oxidation state and sulfurized CoMo/Al_2_O_3_ catalysts using Al_2_O_3_-Spongy and Al_2_O_3_-PB as support.

After the loading of Co and Mo on Al_2_O_3_-Spongy and Al_2_O_3_-PB, powder XRD patterns for oxidation state CoMo/Al_2_O_3_ supported catalyst (denoted as Catalyst-Spongy-O and Catalyst-PB-O accordingly) were recorded to confirm the phase and dispersion of active metal sites on different alumina support. The XRD patterns of oxidation state catalysts are shown in Figure 5. For Catalyst-PB-O, in addition to the characteristic diffraction peaks of γ-Al_2_O_3_, reflections at 22.2° and 23.3° arise from the phase of Al_2_(MoO_4_)_3_ (JCPDS 00-020-0034), while the diffraction peaks at 26.5° and 32.1° are attributed to the phase of CoMoO4 (JCPDS 00-021-0868). This result suggests that except for the formation of CoMoO_4_, some of the deposited MoO_3_ interacted with Al_2_O_3_-PB and transformed into Al_2_(MoO_4_)_3_ on the surface of support during the calcination, implying a strong interaction between Mo oxide species and Al_2_O_3_-PB. In contrast, when using Al_2_O_3_-Spongy as support, no diffraction peaks of active phase in oxidation state can be detected, indicating the improved dispersion of Mo species. Combined with the structure and pore properties of Al_2_O_3_-Spongy, it is believed that the MoO_3_ and CoMoO_4_ are highly dispersed on the surface of support, which is mainly because the thin alumina lamina in spongy-like structure could provide higher surface area and expose enough sites to immobilize the active components. Because of the low loading of CoO, no characteristic peaks of CoO were observed in the patterns of oxidation catalyst.

Prior to hydrodesulfurization (HDS) reaction evaluation, fresh oxidation state catalysts need to be presulfurized, and the corresponding XRD patterns of sulfurized state catalysts are shown in Figure 5. It appears that the original typical diffraction peaks attributing to Al_2_(MoO_4_)_3_ and CoMoO_4_ phases disappear in the XRD patterns of the sulfided Catalyst-PB-S, indicating sulfurization of the oxidation state active phase. Besides, each pattern presents low characteristic diffraction peaks at 32.9° and 58.2° corresponding to (101) and (110) crystal plane in MoS_2_ (JCPDS 00-037-1492), suggesting that the formed MoS_2_ possesses a poorly crystalline structure with small slab size. In addition, no XRD peaks of CoMoS phase were found on patterns of sulfided catalyst, illustrating that the superficial CoMoS species are in good dispersion, either in the form of amorphous particles or in small crystallites (≤4 nm) which cannot be detected by XRD.

**Figure 6:**
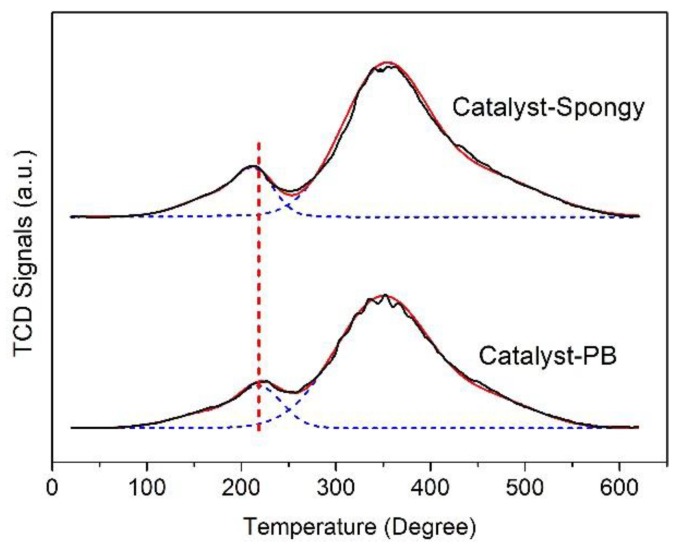
TPR profiles of the sulfurized CoMo/Al_2_O_3_ catalysts using different support.

The H_2_-TPR patterns of sulfurized CoMo/Al_2_O_3_ catalysts were presented in Figure 6. It could be seen that the H_2_-TPR spectra present two reduction peaks in the range of 70–570 °C. The peak at low-temperature around 210 °C can be assigned to the reducible surface sulfur atoms that are weakly bonded to Mo, which determines the number of vacant sites available during HDS [31,32], the other broad peak at about 350 °C corresponds to the partial reduction of the small MoS_2_ crystallites [33]. It was believed that reducing the surface sulfur atoms at low temperature could create coordinative unsaturated sites (CUS), and these formed sulfur vacancies are believed to be the catalytic sites [31]. The reduction temperature of the first peak in Catalyst-Spongy-S decreased to 210 °C compared with Catalyst-PB-S (219 °C), which indicates weakened interactions of Mo-S bridge and the existence of surface sulfur atoms with high activity on the surface of Catalyst-Spongy-S. The reduction temperatures of the peaks at high temperature did not notably change, suggesting that the two alumina support did not obviously affect the activity of small MoS_2_ crystallites. From this viewpoint, it is thought that the HDS catalyst using Al_2_O_3_-Spongy as support would exhibit higher HDS activity among the two catalysts.

**Figure 7:**
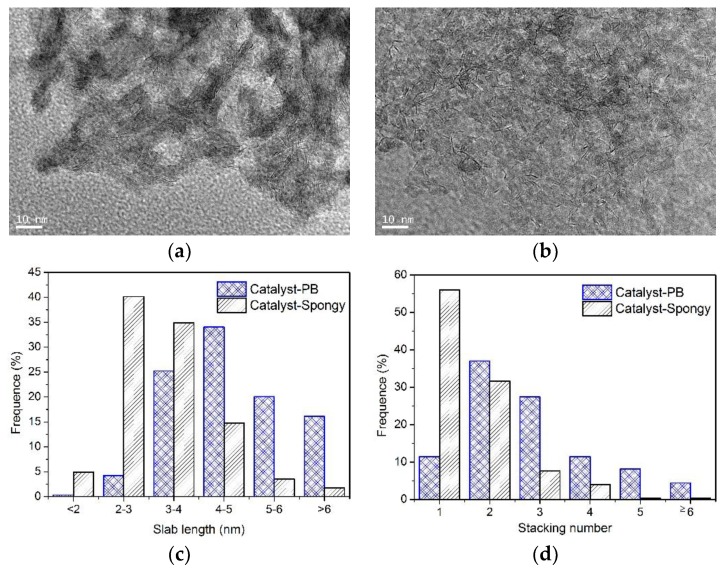
HRTEM images of sulfurized catalysts: (**a**) Catalyst-PB-S, (**b**) Catalyst-Spongy-S, and distributions of (**c**) slab length and (**d**) stacking number of MoS_2_ slabs on sulfurized CoMo/Al_2_O_3_ catalysts.

To acquire information about the dispersion and morphology of active phase, HRTEM micrographs of the presulfurized catalysts are shown in Figure 7a,b. The black fringes in the images were indexed to the (002) basal planes of MoS_2_ crystallites, and the interlayer distances were about 0.62 nm. Considering that the morphology and orientation of MoS_2_ phase have a close relationship with the HDS activity of catalysts [23], the statistical distribution of MoS_2_ slab lengths and stacking numbers were presented in Figure 7c,d, and the average layer length, average stacking number, as well as dispersion degree of MoS_2_ slabs are listed in Table 3. When using Al_2_O_3_-Spongy as support, the length of the MoS_2_ slabs were mainly concentrated between 2–4 nm, while the slabs over Catalyst-PB-S presents relatively longer slabs that are mostly in the range of 3–6 nm. As for stacking number, the MoS_2_ slabs of Catalyst-Spongy-S exhibited a higher frequency of one and two layers, whereas the highest frequency of MoS_2_ slabs with 2 and 3 layers was observed in the Catalyst-PB-S, suggesting that the spongy-like microstructure of Al_2_O_3_-Spongy could bring about better dispersion of the active phase. The lower stacking degree and smaller slab length of Catalyst-Spongy-S could ascribe to the high surface area and more weak Lewis acid sites on the surface of spongy-like support. When the precursor of the active phase comes into contact with the alumina support, the exposed weak Lewis acid sites could interact and fix the precursor on the alumina surface, and the interactions between Lewis acid sites and active phase prevent the increase of stacking number.

**Figure 8:**
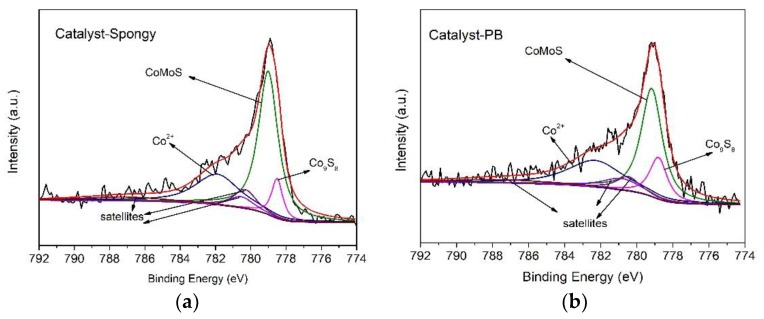
The measured and curve-fitted Co2p XPS spectra of (**a**) Catalyst-Spongy-S and (**b**) Catalyst-PB-S.

The XPS of sulfurized catalysts were performed to probe the incorporation of Co within MoS_2_ phase. In the high-resolution scans and corresponding fitting curves of the Co2p XPS spectra in Figure 8, the peaks at 778.8 eV in the Co2p deconvolution spectra are attributed to the CoMoS phases; the binding energies at 779.2 eV are ascribed to the Co_9_S_8_ species; those at 782.3 eV are ascribed to Co^2+^ species; and the three broaden peaks are the satellite signals. The content of different Co species on the sulfurized catalysts are listed in Table 4. The reference Catalyst-PB-S presents lower percentage of cobalt in CoMoS species and higher percentage of Co_9_S_8_ species on the surface. When using Al_2_O_3_-Spongy as support, the relative content of CoMoS species increased at Catalyst-Spongy-S, indicating more Co atoms incorporated with MoS_2_ and forming active CoMoS species. Since Co_9_S_8_ is less active in HDS reaction, Catalyst-Spongy may presents higher activity than Catalyst-PB.

### 3.3. Hydrodesulfurization Activity

**Figure 9:**
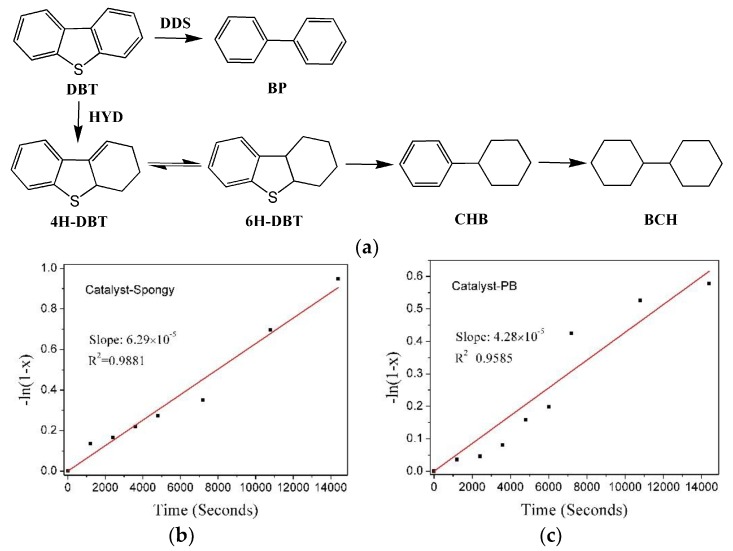
(**a**) The HDS network of DBT over hydrotreating catalysts. DBT (dibenzothiophene), BP (biphenyl), CHB (cyclohexylbenzene), BCH (bicyclohexyl), 4H-DBT (tetrahydrodibenzothiophene), 6H-DBT (hexahydrodibenzothiophene). Linear regression fit of −ln(1 − x) against time plots for (**b**) Catalyst- Spongy-S and (**c**) Catalyst-PB-S when the conversion of DBT is below 40%.

To compare the effect of support on the catalytic performance of HDS catalysts, the HDS catalyst evaluation was carried out in batch reactor using dibenzothiophene (DBT) as model compounds. Reaction pathways for DBT transformation are shown in Figure 9a, and this process can be achieved through two routes: direct desulfurization (DDS) and hydrodesulfurization (HYD). The DDS route proceeded via direct hydrogenolysis of the C-S bonds, while in HYD route the prehydrogenation of the aromatic rings in the sulfur compounds preceded sulfur extrusion [34,35]. The HDS of DBT through two parallel reaction pathways leads to different products, biphenyl (BP) along DDS route, and cyclohexylbenzene (CHB) and bicyclohexyl (BCH) along HYD route; therefore, the ratio of different products reflect the selectivity of two reaction pathways.

By comparison, the results of conversion, initial HDS rate constants k_HDS_ (expressed in mol Kg_cat_^−1^ s^−1^, assuming pseudo-first-order kinetics) and the TOFs for different catalysts during the HDS of DBT are listed in Table 5. With regard to activity results, it is shown that Catalyst-Spongy-S apparently exhibits higher HDS activity than Catalyst-PB-S, and 93.5% of DBT was desulfurized by Catalyst-Spongy-S, which is much higher than that of Catalyst-PB-S. The overall k_DBT_ were determined from the slope of corresponding −ln(1 − x) against time plots shown in Figure 9b,c. The as-synthesized Catalyst-Spongy-S indeed presents higher k_HDS_ value of HDS reaction (18.8 × 10^−^^5^ mol Kg_cat_^−1^ s^−1^), 47% higher than that of Catalyst-PB-S (12.8 × 10^−^^5^ mol Kg_cat_^−1^ s^−1^). The enhanced k_DBT_ of Catalyst-Spongy-S mainly attributes to the high dispersion of active phase, which could expose more active sites. Besides, Catalyst-Spongy-S also presents much higher TOF value than Catalyst-PB-S. This result is in accordance with TPR data, showing that the catalyst with more active reducible sulfur species possesses relatively higher HDS activity.

Table 5 also shows the reaction product distributions of the two catalysts at the similar DBT conversion (about 40%); the contributions of DDS and HYD routes to the overall HDS performance were separated. Selectivity ratios of HYD/DDS over catalysts are within 1, indicating that the HDS proceeds predominantly over the DDS route. However, compared with Catalyst-PB-S, the product distributions change with the variation of catalyst support. For Catalyst-Spongy-S, the proportion of CHB and 4H-DBT are higher than that of Catalyst-PB-S, indicating that the HYD route is promoted to the DDS route because of the high distribution of active phase and changed morphology of MoS_2_ nanoparticles.

The above activity assessment results show that the microstructure of support could affect the morphology of the active phase, which finally influences the HDS activity and selectivity of reaction route. In terms of the rim-edge model [36,37], the location of HYD sites is limited on the top and bottom slab of the MoS_2_ crystallites (rims), while the sites responsible for direct desulfurization are located on all slabs side of a MoS_2_ stack (edges and rims). Since the HYD/DDS ratio is closely related to the ratio of rim/edge, the stacking degree and slab length of MoS_2_ slabs could modulate the selectivity of HDS in DBT [38]. In Catalyst-PB-S, the crystallites of the active phase are multi-layer with long linear size, and this system is accompanied by more edge sites, which is favorable for higher DDS activity. For Catalyst-Spongy-S, the laminar alumina in spongy-like structure improves the dispersion of the metal precursors, and decreases the average slab length and stacking layers of (Co)MoS_2_ slabs, resulting in higher exposure ratio of rims and promoted HYD selectivity. DBT mainly exists in diesel, and decreasing the mass ratio of aromatic hydrocarbon is an important factor to improving diesel quality; therefore, the promoted HYD selectivity is beneficial for quality upgrading of diesel oil.

The high dispersion of the active phase and improved activity of Catalyst-Spongy-S can be attributed to the structure of Al_2_O_3_-Spongy. Compared with the stacked spherical particles in Al_2_O_3_-PB, the thin laminar in spongy-like structure affords more available exterior surface, which could expose more acidic sites and provide more anchoring sites for the active phase, resulting in the high dispersion of (Co)MoS_2_ phases on Al_2_O_3_-Spongy. The highly dispersed active phase on Catalyst-Spongy-S could offer more coordinated unsaturation edges and rim sites. Therefore, Catalyst-Spongy-S presents different catalytic activity from industrial alumina due to the spongy-like support that provides more vacancies and surface area to anchor the active phase, which changes the morphology of well-dispersed (Co)MoS_2_ active phases, as evidenced by the TPR, HRTEM results.

## 4. Conclusions

Herein, a kind of γ-alumina (γ-Al_2_O_3_) with spongy-like structure was prepared by two-step pyrolysis of Al-based metal-organic frameworks (noted Al_2_O_3_-Spongy). Compared with γ-Al_2_O_3_ from commercial pseudo-boehmite (noted Al_2_O_3_-PB), the as-synthesized spongy alumina presents higher surface area, bimodal pore size distribution, larger pore volume, and more acidic sites. Therefore, Al_2_O_3_-Spongy possesses great advantages as a catalyst support for CoMo/Al_2_O_3_ catalysts in the hydrodesulfurization (HDS) of dibenzothiophene (DBT). Among the two catalysts using Al_2_O_3_-Spongy and Al_2_O_3_-PB as support, Catalyst-Spongy-S shows highly dispersed (Co)MoS_2_ active phase, more active surface sulfur species, and higher catalytic performance under the same reaction conditions (T = 300 °C, P = 7.2 MPa). Because of the low stacked layers and shorter slab length in Catalyst-Spongy-S, the active phase could expose more coordinative unsaturation rim and edge sites, which is favorable for the HDS reaction, resulting in higher DBT conversation than Catalyst-PB-S. Besides, since the rim sites are responsible for both direct desulfurization (DDS) and hydrodesulfurization (HYD) routes (whereas the edge sites only favor DDS routes), the higher rim/edge ratio changes the products’ selectivity and produces more HYD products. Our study provides a new insight into the rational design and fabrication of mesoporous alumina with different microstructures using metal-organic frameworks, which will offer more choices and promote the development of highly active HDS catalysts.

## Figures and Tables

**Table 1:** Chemical analysis of the obtained alumina samples.

Sample	Al_2_O_3_ Content/%	Contents of Impurity (%)		
		Na_2_O	Fe_2_O_3_	SiO_2_
Al_2_O_3_-Spongy	>99.9	0.026	0.002	0.009
Al_2_O_3_-PB	99.67	0.304	0.009	0.016

**Table 2:** Textural properties of the obtained alumina samples.

Sample	Specific Surface Area [m^2^ g^−1^]	Pore Volume [cm^3^ g^−1^]	Average Pore Size [nm]
Al_2_O_3_-Spongy	350	0.63	7.2
Al_2_O_3_-PB	240	0.52	8.6

**Table 3:** Average length ($\bar{L}$), stacking layer numbers ($\bar{N}$) and fraction of Mo atoms on the edge surface (*f_Mo_*) in sulfided catalysts.

Sample	$\bar{L}$ [nm]	$\bar{N}$	*f_Mo_*
Catalyst-PB-S	4.78	2.81	0.25
Catalyst-Spongy-S	3.29	1.62	0.35

**Table 4:** XPS analysis results of the different contributions of Co2p to Catalyst-Spongy-S and Catalyst-PB-S.

Sample	CoMoS		Co_9_S_8_		Co^2+^	
	B.E. (eV)	At %	B.E. (eV)	At %	B.E. (eV)	At %
Catalyst- Spongy-S	779.0	61.1	778.5	9.7	781.9	29.2
Catalyst-PB-S	779.2	50.7	778.8	16.4	782.3	32.9

**Table 5:** The HDS conversion, initial reaction rate constant k_HDS_, TOF and product selectivity of HDS catalysts at 300 °C.

Sample	Conversion ^1^ [%]	k_HDS_^2^/[10^−5^ mol Kg_cat_^−1^ s^−1^]	TOF ^3^ [h^−1^]	Product Selectivity ^4^ [%]			HYD/DDS Selectivity ^4^
				BP	CHB	4H-DBT	
Catalyst-PB-S	82.2	12.8	1.10	81.4	17.6	1.0	0.229
Catalyst-Spongy-S	93.5	18.8	3.07	71.6	28.0	0.4	0.397

^1^ This is the final HDS conversion of DBT at 300 °C for 8 h. ^2^ Initial reaction rate constant k_HDS_ was obtained when the total DBT conversion is below 40%. ^3^ Number of reacted DBT molecules per hour on per Mo atom at the edge surface is obtained at about 20% of total DBT conversion. ^4^ The product distribution and HYD/DDS selectivity of catalysts were calculated when the total DBT conversion was about 40%.
